# Magnetic resonance imaging and clinical prediction of intracranial atherosclerotic large vessel occlusion in acute ischemic stroke treated with endovascular thrombectomy

**DOI:** 10.3389/fneur.2026.1803264

**Published:** 2026-05-22

**Authors:** Yohei Tateishi, Hiroaki Otsuka, Aya Yamashita, Keiichiro Onizuka, Takanori Amano, Daiji Torimura, Yuki Tomita, Takuro Hirayama, Tomoaki Shima, Shunsuke Yoshimura, Teiichiro Miyazaki, Yuki Matsunaga, Hajime Maeda, Akira Tsujino

**Affiliations:** 1 Department of Neurology and Strokology, Nagasaki University Hospital, Nagasaki, Japan; 2 Unit of Clinical Medicine, Department of Clinical Neuroscience, Nagasaki University Graduate School of Biomedical Sciences, Nagasaki, Japan; 3 Department of Neurosurgery, Nagasaki University Graduate School of Biomedical Sciences, Nagasaki, Japan

**Keywords:** acute ischemic stroke, anterior cerebral artery, atrial fibrillation, intracranial atherosclerosis, magnetic resonance imaging

## Abstract

**Introduction:**

Intracranial atherosclerosis-related large-vessel occlusion (ICAS-LVO) poses specific challenges during endovascular thrombectomy, and its early identification is clinically important for efficient recanalization.

**Methods:**

This retrospective, singlecenter study included consecutive patients who underwent magnetic resonance imaging (MRI) and endovascular thrombectomy for anterior circulation LVO. The candidate predictor set included predefined MRI markers and clinical variables, including atrial fibrillation, as documented in medical history or electrocardiogram. Variable importance was assessed using a random forest classifier, and the most informative MRI and clinical predictors were then entered into multivariable logistic regression to derive the point-based score. ICAS-LVO discrimination was quantified using the area under the receiver operating characteristic curve (AUC) and internally validated via bootstrap resampling.

**Results:**

Among 335 eligible patients, 44 (13%) had ICAS-LVO. In the multivariable analysis, absence of atrial fibrillation (odds ratio [OR], 20.14; 95% confidence interval [CI], 6.02–89.89; *p* < 0.001), multiple cortical/border-zone infarcts (OR, 10.96; 95% CI, 3.65–34.90; *p* < 0.001), mixed acute–subacute lesions (OR, 5.10; 95% CI, 1.51–17.84; *p* = 0.009), and absence of a susceptibility vessel sign (OR, 4.63; 95% CI, 1.81–12.51; *p* = 0.002) were independently associated with ICAS–LVO. The resulting ICAS-M score demonstrated high discrimination for ICAS-LVOs in this single-center cohort with 44 ICAS-LVO events (AUC 0.940), which was preserved during internal validation.

**Conclusion:**

In an MRI-capable thrombectomy center, the ICAS-M score demonstrated high, internally validated discrimination for ICAS-LVO and may support the rapid planning of ICAS-tailored endovascular strategies.

## 1 Introduction

Intracranial atherosclerotic disease is emerging as the principal cause of acute ischemic stroke in diverse populations, particularly in East Asia and among Black and Hispanic individuals (1). Intracranial atherosclerosis-related large vessel occlusion (ICAS-LVO) presents distinctive procedural challenges compared with embolic etiologies, including lower first-pass recanalization rates, more frequent reocclusion, and a greater need for rescue interventions such as angioplasty and stenting (2). Therefore, early ICAS-LVO identification could lead to efficient recanalization through the selection of appropriate procedural strategies and adjunctive antithrombotic therapies (3, 4).

Recent approaches have incorporated computed tomography (CT) angiography and clinical risk factor models to predict ICAS-LVO occurrence (5–10). However, implementing these models may require time-consuming assessment due to the need for detailed clinical histories and calculation of composite scores. Magnetic resonance imaging (MRI) has been demonstrated to be more sensitive than CT for detecting early acute ischemic lesions (11). Moreover, the European Stroke Organization has proposed watershed infarction and the absence of susceptibility vessel signs (SVS) on MRI as key imaging findings for predicting ICAS-LVO (12). Although these observations highlight the potential value of MRI-based assessment, only a few studies have developed MRI-based prediction models for ICAS-LVO, and concerns remain regarding the feasibility of MRI in all time-sensitive thrombectomy pathway (13).

This study aimed to develop a simple MRI- and clinical-based predictive score for ICAS-LVOs that combined readily assessable lesion patterns with key clinical characteristics in patients treated with endovascular thrombectomy.

## 2 Methods

### 2.1 Study design and setting

This retrospective, single-center observational study was conducted at Nagasaki University Hospital. In this tertiary stroke center, MRI is routinely used as a first-line imaging modality for suspected acute ischemic stroke.

### 2.2 Patient selection

Consecutive patients with acute ischemic stroke who underwent endovascular thrombectomy between January 2016 and September 2025 were included in the study. Eligible patients had emergent LVO of the internal carotid artery terminus or the M1 or proximal M2 segments of the middle cerebral artery, as confirmed by MR angiography, and underwent mechanical thrombectomy. We excluded patients who underwent only CT at presentation due to contraindications to MRI (such as non–MRI-compatible metallic implants) or in whom MRI was not feasible because of severe motion artifacts or unstable clinical conditions. We included patients with active cancer, defined as a malignancy diagnosed or recurrent within 6 months or requiring ongoing anticancer treatment at presentation, because detailed information on cancer status is often difficult to obtain in the emergency setting. Moreover, we included patients initially diagnosed with acute ischemic stroke by MRI at referral hospitals who were transferred directly for thrombectomy without repeat MRI; these patients were classified as having undergone an “outside-hospital MRI.” Baseline demographics, vascular risk factors, National Institutes of Health Stroke Scale (NIHSS) scores, time metrics, and intravenous thrombolysis use were obtained from the medical records.

### 2.3 Diagnosis of atrial fibrillation (AF)

The presence of atrial fibrillation (AF) in the emergency department was defined as a documented AF history or AF detected by electrocardiographic monitoring or 12-lead electrocardiogram.

### 2.4 MRI acquisition and assessment

At our hospital, MRI was performed on 1.5-T scanners using a standardized acute stroke protocol, including axial diffusion-weighted imaging (DWI), fluid-attenuated inversion recovery (FLAIR), MR angiography, and susceptibility-weighted imaging (SWI) with 5-mm slices. Based on previous reports, three MRI markers were evaluated as candidate ICAS-LVO indicators: (1) multiple cortical/border–zone infarcts, defined as ≥5 small acute infarcts within the cortical and/or subcortical territory of the relevant artery, including internal and cortical border–zone infarcts (14, 15), an *a priori* threshold chosen based on clinical experience to represent a clearly disseminated lesion pattern; (2) mixed acute-subacute infarcts, defined as the coexistence, within the same vascular territory, of at least one DWI-hyperintense lesions without corresponding FLAIR hyperintensity and at least one hyperintense lesion on both DWI and FLAIR (16); and (3) absence of a SVS on SWI or T2* at the occlusion site (17). Ischemic lesion size was assessed using DWI-ASPECTS. Ischemic involvement in each DWI-ASPECTS subregion (caudate, lentiform nucleus, internal capsule, insula, and cortical regions M1–M6) was scored and dichotomized as present or absent, and the scan duration was recorded. For patients who had MRI at outside hospitals, the core sequences for this study (DWI, FLAIR, and SWI or T2*-weighted imaging) were required to be available with axial slice thickness ≤ 5 mm and sufficient image quality for reliable assessment, as judged by the study neurologists. Patients without these core sequences or with image quality insufficient for reliable assessment were excluded. In March 2025, we introduced a streamlined MRI protocol that incorporated the same core sequences to reduce the scanning duration. All MRI scans were reviewed independently by two board-certified stroke neurologists (A. Y. and T. M.) blinded to the angiographic classification, and disagreements were resolved by consensus. For the predefined MRI predictors of interest, inter-rater agreement for each marker was assessed using Cohen’s kappa coefficient.

### 2.5 ICAS-LVO definition

ICAS-LVO was defined angiographically during endovascular thrombectomy. Following reperfusion procedures, ICAS-LVO was identified when the target artery exhibited persistent focal residual stenosis exceeding 50% at the original occlusion site or early reocclusion at the same location despite initial recanalization (18, 19).

### 2.6 Statistical analysis and model development

Because there were no missing data for the predictors or the outcome, we performed all analyses on complete data without imputation. Continuous and categorical variables are reported as medians with interquartile ranges (IQR) and as counts and percentages, respectively. Group comparisons between ICAS-LVO and non–ICAS-LVO were performed using Fisher’s exact test for categorical variables and the Mann–Whitney *U* test for continuous variables. Baseline characteristics were compared between groups using standard univariable tests. These comparisons were considered exploratory. Therefore, *p*-values were not adjusted for multiple testing and should be interpreted descriptively. To explore the factors associated with ICAS-LVO, univariate analyses were performed on the clinical and MRI variables. Because the number of ICAS-LVO events was relatively limited, including all significant variables in a conventional multivariable logistic regression model, was considered to carry a high overfitting risk. Therefore, we first applied a random forest classifier to all available clinical and MRI variables to rank their relative importance and screen for the candidate predictors, with variable importance estimated using the mean decrease in Gini impurity. Based on this ranking and clinical interpretability, we selected a small set of candidate predictors and fitted multivariable logistic regression models, including these variables, to estimate their odds ratios and regression coefficients. Using the regression coefficients from the final logistic model, we derived a simple points-based score by assigning integer weights to the selected predictors in proportion to the magnitude of their coefficients. We assessed the discrimination of this score for ICAS-LVO using the area under the receiver operating characteristic (ROC) curve (AUC). To identify a single operating threshold of this score, we determined the cut-off that maximized the Youden index (sensitivity + specificity − 1) on the ROC curve. We compared the AUC of the point-based score with those of the individual predictors using DeLong’s test for two correlated ROC curves. Internal validation was performed using bootstrap optimism correction with 1,000 resamples. Model calibration was evaluated by estimating the calibration intercept and slope from a logistic calibration model and by visually inspecting calibration plots based on deciles of predicted risk. We did not incorporate time metrics into the multivariate analysis, because these variables depend on country-specific geography and local workflow organization. This incorporation may compromise the external validity of a simple score intended for use in diverse MRI-capable centers. To reduce small-sample bias, we fitted a Firth penalized logistic regression model, including the same predictors as in the main multivariable model. As separate sensitivity analyses, we repeated the evaluation of the prediction score in two restricted cohorts: the patients whose MRI examinations were performed at our hospital (excluding outside-hospital MRI) and patients without active cancer. In each analysis, we calculated the AUC of the predicting score in the same manner as in the primary analysis. Statistical significance was defined as a two-sided *p*-value of <0.05. All analyses were performed using JMP version 19 (JMP Statistical Discovery LLC, SAS, Cary, NC, United States) and RStudio software (Posit PBC, Boston, MA, United States).

### 2.7 Ethical considerations

The institutional review board of our hospital approved the study protocol and waived the requirement for written informed consent due to the retrospective design and the use of anonymized data, in accordance with national regulations and the Declaration of Helsinki.

## 3 Results

**Figure 1:**
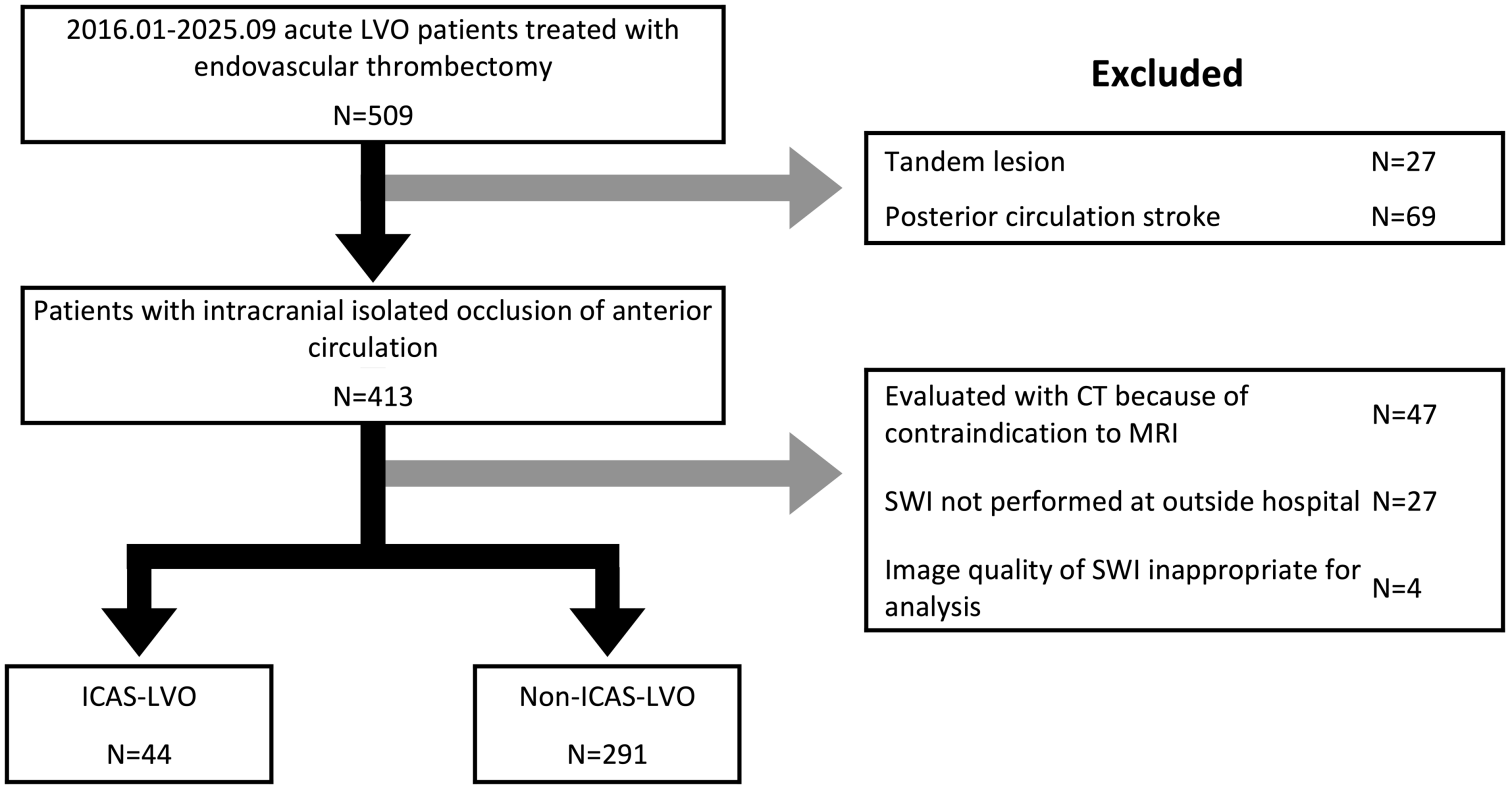
Flow diagram of patient selection. LVO, large vessel occlusion; ICAS, intracranial atherosclerosis; CT, computed tomography; SWI, susceptibility-weighted imaging.

Overall, 509 patients underwent endovascular thrombectomy for acute ischemic stroke. After applying the prespecified exclusion criteria, 335 patients (44 with ICAS-LVO and 291 with non–ICAS-LVO) were included in the final analysis (Figure 1).

The baseline clinical and imaging characteristics are summarized in Table 1. Compared with the non-ICAS-LVO group, patients with ICAS-LVO tended to be younger, have a lower AF prevalence, a higher prevalence of atherosclerotic risk factors (including diabetes, dyslipidemia, and smoking), and lower baseline NIHSS scores; these univariable comparisons are exploratory and unadjusted for multiple testing.

**Table 1:** Baseline characteristics of patients in the ICAS-LVO and non-ICAS-LVO groups.

	All patients		ICAS-LVO		Non-ICAS-LVO		*p*-value
	*N* = 335		*N* = 44		*N* = 291		
Sex: M/F	152/183		24/20		128/163		0.198
Age	80	(72–87)	76	(64–82)	81	(73–87)	0.001
Pre-stroke mRS ≤ 2	272	(81)	41	(93)	231	(80)	0.036
Vascular risk factors							
Hypertension	264	(79)	38	(86)	226	(78)	0.237
Diabetes mellitus	56	(17)	13	(30)	43	(15)	0.028
Dyslipidemia	88	(26)	20	(45)	68	(23)	0.003
Atrial fibrillation at presentation	182	(54)	4	(9)	178	(61)	< 0.001
Smoking	44	(13)	14	(32)	30	(10)	0.001
Past medical history							
Ischemic heart disease	25	(7)	4	(9)	21	(7)	0.553
Ischemic stroke	57	(17)	2	(5)	55	(19)	0.017
Active cancer	24	(7)	2	(5)	22	(8)	0.753
Clinical presentation							
Baseline NIHSS score	18	(13–22)	12	(7–17)	18	(14–23)	< 0.001
Imaging findings							
DWI-ASPECTS	7	(6–9)	8	(7–9)	7	(6–9)	0.001
Caudate	92	(27)	10	(23)	82	(28)	0.587
Lentiform	162	(48)	23	(52)	139	(48)	0.629
Internal capsule	15	(4)	1	(2)	14	(5)	0.703
Insular cortex	195	(58)	11	(25)	184	(63)	< 0.001
M1	120	(36)	4	(9)	116	(40)	< 0.001
M2	105	(31)	8	(18)	97	(33)	0.054
M3	36	(11)	8	(18)	28	(10)	0.113
M4	56	(17)	3	(7)	53	(18)	0.080
M5	153	(46)	10	(23)	143	(49)	0.001
M6	41	(12)	7	(16)	34	(12)	0.458
Multiple cortical/border-zone infarcts	54	(16)	32	(73)	22	(8)	< 0.001
Mixed acute-subacute infarcts	39	(12)	24	(55)	15	(5)	< 0.001
Absence of SVS	96	(29)	26	(59)	70	(24)	< 0.001
Occlusion site							0.084
Intracranial ICA	91	(27)	6	(14)	85	(29)	
M1 segment of MCA	185	(55)	30	(68)	155	(53)	
M2 segment of MCA	59	(18)	8	(18)	51	(18)	
Acute treatments							
Intravenous thrombolysis	150	(45)	16	(36)	134	(46)	0.257

Atrial fibrillation at presentation: documented atrial fibrillation on pre-stroke history or on admission electrocardiogram.

mRS, modified Rankin scale; NIHSS, National Institutes of Health Stroke Scale; DWI, diffusion–weighted imaging; ASPECTS, Alberta Stroke Program Early CT Score; SVS, susceptibility vessel sign; ICA, Internal carotid artery; MCA, middle cerebral artery.

Supplementary Table 1 presents information regarding the stroke subtypes and their respective imaging, time metrics, and clinical outcomes. Onset-to-door, onset-to-puncture, and puncture-to-recanalization times, were generally longer in the ICAS-LVO group than in the non-ICAS-LVO group. The MRI scan duration decreased from a median of 17 min (IQR, 16–20) before March 2025 to 10 min (IQR, 9–14) (*p* < 0.001). Concurrently, the door-to-puncture time decreased from 69 min (IQR, 57–81) to 56 min (IQR, 43–71; *p* = 0.002).

On admission MRI, patients with ICAS-LVO had higher DWI-ASPECTS scores and were more likely to exhibit multiple cortical/border–zone infarcts, mixed acute-subacute infarcts, and absence of SVS (Table 1). The insula, M1, and M5 regions were less frequently involved than in the non-ICAS-LVO group. In the inter-rater agreement analysis, the *κ* values (95% confidence interval [CI]) for multiple cortical/border-zone infarcts, mixed acute-subacute infarcts, and absence of SVS were 0.818 (0.736–0.900), 0.744 (0.637–0.850), and 0.792 (0.637–0.850), respectively, indicating good agreement for all three MRI markers.

**Figure 2:**
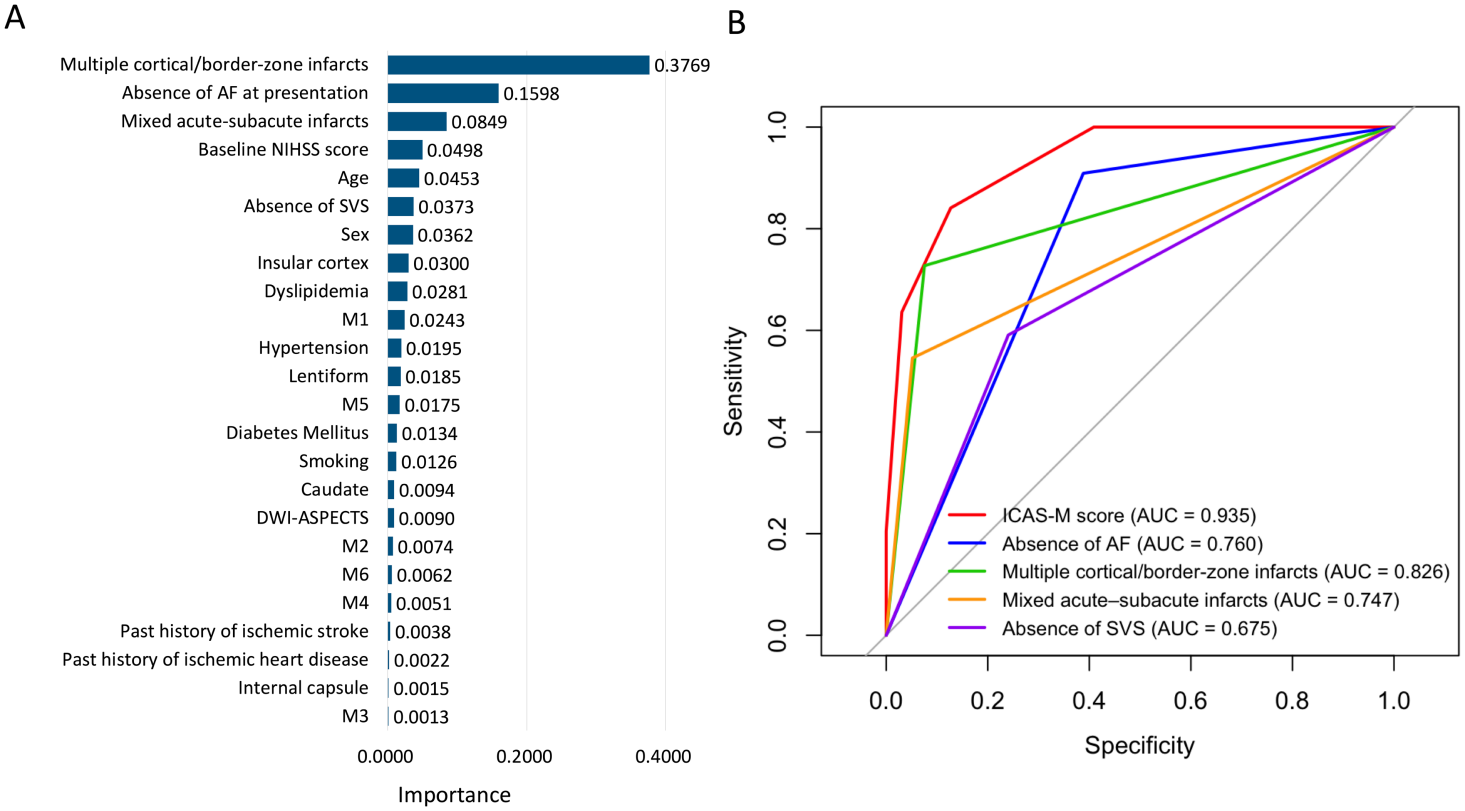
**(A)** Feature importance ranking based on the random forest algorithm. **(B)** Comparison of receiver operating characteristic curves and AUCs for the ICAS-M score, absence of AF, multiple cortical/border-zone infarcts, mixed acute-subacute infarcts, and absence of SVS. AUCs were compared using DeLong’s test for correlated receiver operating characteristic curves. AUC, area under the curve; AF, atrial fibrillation; SVS, susceptibility vessel sign.

**Figure 3:**
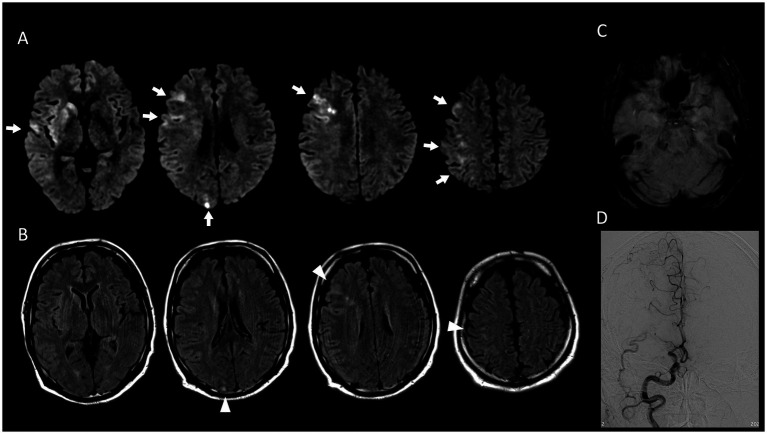
A representative case with high ICAS-M score. DWI (*b* = 1,000 s/mm^2^) revealed multiple cortical/border-zone infarcts in the right middle cerebral territory (white arrows, **A**). Corresponding FLAIR images of the same slices demonstrate mixed acute-subacute infarcts within the same vascular territory (white arrowheads, **B**). The DWI-ASPECT score was 5, indicating infarction in the caudate, lentiform, M2, M3, and M5 regions. Susceptibility-weighted imaging (gradient-echo sequence, slice thickness 5 mm) shows no susceptibility vessel sign in the right M1 segment **(C)**. Digital subtraction angiography confirms occlusion of the right middle cerebral artery **(D)**. DWI, diffusion-weighted imaging; FLAIR, fluid-attenuated inversion recovery; ASPECTS, Alberta Stroke Program Early CT score.

In the random forest model, multiple cortical/border-zone infarcts, absence of AF, mixed acute–subacute lesions, baseline NIHSS score, age, and absence of SVS were informative ICAS-LVO predictors (Figure 2A). Based on variable importance and the need for rapid, reproducible bedside assessment, we constructed a four-item MRI- and rhythm-based ICAS-M score comprising multiple cortical/border-zone infarcts, absence of AF, mixed acute–subacute infarcts, and absence of SVS. We termed this model the ICAS-M score, with “M” indicating the “MRI-based” lesion patterns (“Multiple” cortical/border–zone and “Mixed” acute–subacute infarcts) used in the score. In the final multivariable logistic model, absence of AF showed a larger regression coefficient than the three imaging predictors (Table 2). To construct the ICAS-M score, we assigned 2 points to absence of AF and 1 point to each imaging predictor; therefore, the point-based score reflected the relative magnitude of the regression coefficients while remaining simple to apply at the bedside (range, 0–5 points). A representative example is displayed in Figure 3 and Supplementary Figure 1. The ICAS-M score discriminated between the ICAS-LVO and non–ICAS-LVO groups, with an AUC of 0.935 (95% CI, 0.904–0.965), which was higher than that of any single predictor, including multiple cortical/border-zone infarcts (AUC 0.826), mixed acute–subacute infarcts (AUC 0.747), absence of SVS (AUC 0.675), and absence of AF (AUC 0.760). In pairwise comparisons, the ICAS-M score achieved higher AUCs than each individual predictor, as confirmed by DeLong’s test for correlated ROC curves (all *p* < 0.001) (Figure 2B). For the integer-valued ICAS-M score, the cut-off that maximized the Youden index corresponded to ≥ 3 points, yielding a sensitivity of 84%, specificity of 87%, positive predictive value of 50%, and negative predictive value of 97% for ICAS-LVO in the derivation cohort. The optimism-corrected AUC obtained from 1,000 bootstrap resamples was 0.939 (95% CI: 0.910–0.969), which was comparable to the apparent AUC. The calibration intercept and slope were approximately 0 and 1, respectively, and the calibration plot based on deciles of predicted risk demonstrated good agreement between predicted and observed ICAS-LVO probabilities (Supplementary Figure 2). The ICAS-M score consistently yielded greater net benefit than treating all or no patients across clinically relevant threshold probabilities, supporting its potential clinical utility in MRI-capable centers (Supplementary Figure 3).

**Table 2:** Predictors of ICAS-LVO.

Predictor	*β* coefficient	OR	95% CI	*p*-value
Absence of AF	1.501	20.14	6.02–89.89	<0.001
Multiple cortical/border–zone infarcts	1.197	10.96	3.65–34.90	<0.001
Mixed acute–subacute infarcts	0.815	5.10	1.51–17.84	0.009
Absence of SVS	0.766	4.63	1.81–12.51	0.002

AF, atrial fibrillation; OR, odds ratio; CI, confidence interval; SVS, susceptibility vessel sign.

Firth’s penalized logistic regression model including the same four predictors demonstrated that the magnitude and direction of the associations were consistent with those from the standard multivariable logistic model (Supplementary Table 2). In additional sensitivity analyses restricted to 308 patients (92%) who underwent MRI at our hospital and 311 patients (93%) without active cancer, the ICAS-M score maintained high discriminatory performance (AUC, 0.945 and 0.949, respectively), similar to that observed in the overall cohort.

## 4 Discussion

In this single-center study of patients undergoing endovascular thrombectomy for anterior circulation LVO, we developed an MRI- and clinical-based ICAS-LVO prediction model (ICAS-M score) incorporating multiple cortical/border–zone infarcts, mixed acute–subacute lesions, absence of SVS, and absence of AF.

In this study, we developed a simple MRI-based score that combines three reproducible lesion patterns with AF status to predict ICAS-LVO before thrombectomy. Several scores have been proposed to predict the occurrence of ICAS-LVO. CT-based scores are based on qualitative or quantitative assessments of the hyperdense artery sign, with inter-rater agreement ranging from fair to moderate (5, 6, 9, 10, 20, 21). Conversely, DWI is more sensitive for hyperacute ischemic lesions and exhibits better inter-rater agreement, with *κ* values around 0.8 compared with approximately 0.5 for CT, even among novices (22, 23). In accordance with these observations, here, the MRI markers incorporated into the ICAS-M score demonstrated adequate inter-rater reliability, indicating that the score may be applied reproducibly, even by physicians with limited stroke imaging experience. The REMIT scale employs a specific MR or CT angiography finding of “non-culprit stenosis.” Additionally, it requires NIHSS score assessment and a biomarker such as B-type natriuretic peptide, which may not be routinely available in all centers (13). Conversely, the ICAS-M score is based solely on routinely obtained acute stroke MRI sequences and simple binary information on the absence of AF at presentation. A direct comparison with existing scores within the same dataset was not performed, and the findings do not establish the superiority of the ICAS-M score over previously proposed models.

Because the ICAS-M score is derived from routinely acquired acute stroke MRI, its applicability is greatest in centers that already implement an MRI-first or MRI-inclusive workflow for thrombectomy triage. Recent evidence on imaging modalities and thrombectomy outcomes is equivocal. A large multicenter study in patients with low-ASPECTS anterior circulation LVO reported no significant differences in reperfusion rates or functional outcomes when patients were selected for thrombectomy using non-contrast CT, CT perfusion, or DWI, suggesting that advanced imaging does not automatically translate into better clinical results (24). Conversely, a systematic review and meta-analysis comparing MRI- versus NCCT-based selection for endovascular therapy within 6 h from onset found higher odds of functional independence, lower rates of symptomatic intracranial hemorrhage, and lower mortality in MRI-selected patients (25). However, these advantages were not observed beyond 6 h. Consequently, these data indicate that MRI-based selection may offer benefits in some early-window settings but is unlikely to be universally superior to CT-based workflows. In this context, the ICAS-M score should be regarded as a complementary tool for centers that already implement MRI in their acute stroke workflow, instead of as a universal argument for adopting an MRI-first triage strategy.

In the random forest analysis, multiple cortical/border–zone infarcts, mixed acute-subacute infarcts, and absence of SVS emerged as informative imaging predictors of ICAS-LVO. To improve inter-rater consistency in the visual assessment, we predefined multiple cortical/border-zone infarcts as the presence of ≥5 small acute lesions within the relevant cortical or subcortical border-zone territory. Clinical variables such as lower NIHSS scores, younger age, male sex, and dyslipidemia were also prominent in the variable importance analysis, supporting an ICAS-related etiology. Importantly, ICAS-LVO has been reported to present with milder neurological deficits, smaller infarct cores, and younger age than cardioembolic LVO (6–8), and dyslipidemia is an established vascular risk factor for atherosclerotic stroke instead of for cardioembolic stroke. Because the baseline NIHSS score depends on detailed neurological examinations and is subject to inter-rater variability, it was not incorporated into the ICAS–M score. Age was also excluded to maintain a simple imaging- and rhythm–based score that could be applied immediately at the time of MRI acquisition and to avoid embedding age distributions that may differ substantially across regions and countries. In our modeling strategy, the random forest classifier was used only to screen and rank candidate predictors, whereas the final ICAS-M score was derived from a multivariable logistic regression model including the selected variables, whose coefficients formed the basis for the point-based score.

The ICAS-M score of 4, where the specificity for ICAS-LVO is high (97%) with the sensitivity of 64%, may help clinicians anticipate the likelihood of underlying ICAS-LVO. Such information may support earlier team preparation and broader decision making regarding antiplatelet regimens and device selection; nonetheless, the optimal antithrombotic protocol likely varies across healthcare systems and requires prospective evaluation (4, 26, 27). The AUC of the ICAS-M score was relatively high for a clinical prediction model, which likely reflects the association between the selected MRI markers and ICAS-LVO in this highly selected, single-center thrombectomy cohort. However, such high performance is expected to attenuate in external settings. Therefore, the ICAS-M score should be considered a preliminary tool that requires external validation and outcome-based prospective testing before it can be used to guide specific procedural or antiplatelet strategies.

This study had certain limitations. First, this study was a retrospective, single-center analysis at an MRI-capable tertiary stroke center with a limited number of ICAS-LVO events, introducing potential overfitting and selection bias; external validation in independent cohorts is required. Second, to limit overfitting we used two-step modeling strategy, first applying random forest to screen candidate predictors and then fitting logistic regression to derive a parsimonious point-based score; because random forest variable importance is not directly comparable to regression coefficients, our variable selection should be regarded as a pragmatic but imperfect approach. In addition, we used the mean decrease in Gini impurity as the importance metric, which is known to be biased toward variables with more categories or higher variance, and we did not systematically compare it with alternative importance measures. Although we explored Firth’s penalized logistic regression model and performed internal validation, and calibration assessment, these effects can only partly mitigate the impact of the limited number of events. In addition, we did not adjust for time metrics, which may act as confounders. Consequently, residual confounding by workflow-related factors cannot be excluded. Third, only patients who underwent MRI were included; therefore, the ICAS-M score was derived in an MRI-eligible thrombectomy population and its generalizability is mainly to centers where acute MRI is routinely available. MRIs from referring hospitals were accepted, and SVS, ASPECTS, and other non-core imaging features were evaluated qualitatively on heterogeneous protocols with 5-mm slices. These images were assessed by two stroke neurologists instead of by multiple neuroradiologists. For the predefined MRI predictors employed in the ICAS-M score, inter-rater agreement was quantified. ASPECTS and other non-core imaging features were evaluated by a single reader without formal reliability assessment, which may limit the precision of these descriptive measures but also reflects the real-world referral networks in which this score would be applied. Fourth, the definition of multiple cortical/border-zone infarcts using a ≥5-lesion threshold was based on clinical judgment instead of prior validation, and dichotomizing this continuous, pattern-based finding may have led to some loss of information. In addition, the mixed acute-subacute infarct pattern was defined qualitatively based on DWI-FLAIR appearances. This approach may partly conflate temporal evolution with spatial hemodynamic heterogeneity, with potential misclassification because of the limited sensitivity of FLAIR in hyperacute or small lesions. Fifth, patients with active cancer were not excluded from the study. Ischemic stroke due to cancer-associated thrombosis often presents with multiple lesions and recurrent infarctions (28, 29). In the time-restricted emergency setting, it is difficult to obtain detailed information regarding the presence and activity of malignancy, which may have led to misclassification. Finally, although the ICAS-M score may support ICAS-tailored endovascular and antiplatelet strategies, periprocedural antithrombotic regimens were not standardized. Moreover, we did not examine whether ICAS-M score-guided management improves reperfusion success or clinical outcomes. In future, these outcome-based effects should be assessed in prospective studies.

## 5 Conclusion

This single-center study developed the ICAS-M score, a simple MRI- and rhythm-based tool that demonstrates high discrimination of ICAS-LVOs in patients undergoing endovascular thrombectomy. Integrating multiple cortical/border-zone infarcts, mixed acute-subacute infarcts, absence of SVS, and absence of AF may facilitate the rapid implementation of ICAS-tailored endovascular strategies in MRI-capable centers.

## Data Availability

The original contributions presented in the study are included in the article/Supplementary material, further inquiries can be directed to the corresponding author.
