# Supplementary material for: Magnetic resonance imaging and clinical prediction of intracranial atherosclerotic large vessel occlusion in acute ischemic stroke treated with endovascular thrombectomy
Source: Front Neurol. 2026 May 22;17:1803264. doi: 10.3389/fneur.2026.1803264 (PMC13236524; doi:10.3389/fneur.2026.1803264)
Supplement: Supplementary file 1 [file Supplementary_file_1.docx]

Supplementary Material

# Supplementary Figures


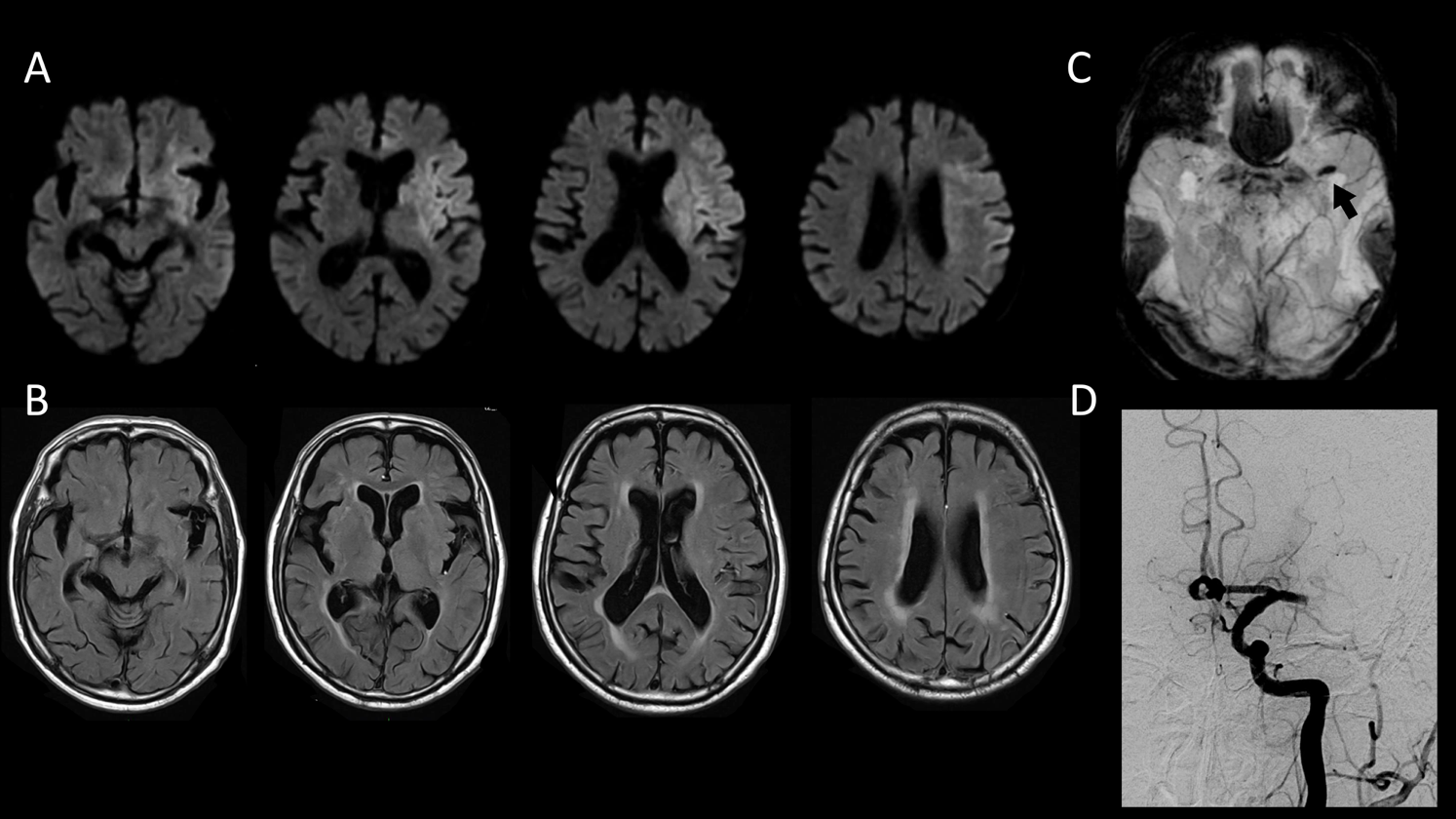


**Supplementary Figure 1.** Representative case with low ICAS-M score

DWI (b = 1000 s/mm^2^) revealed a hyperintense lesion in the left middle cerebral artery territory. The DWI-ASPCETS is 5, indicating infarction in the caudate, lentiform, insular cortex, M1, and M5 regions (A). FLAIR reveals no corresponding hyperintense lesions (B). Susceptibility-weighted imaging (gradient echo sequence, slice thickness 5 mm) demonstrates a susceptibility vessel sign in the left middle cerebral artery (black arrow, C). Cerebral angiography revealed the occlusion of the left middle cerebral artery (D).

DWI, diffusion-weighted imaging; FLAIR, fluid-attenuated inversion recovery; ASPECTS, Alberta Stroke Program Early CT score


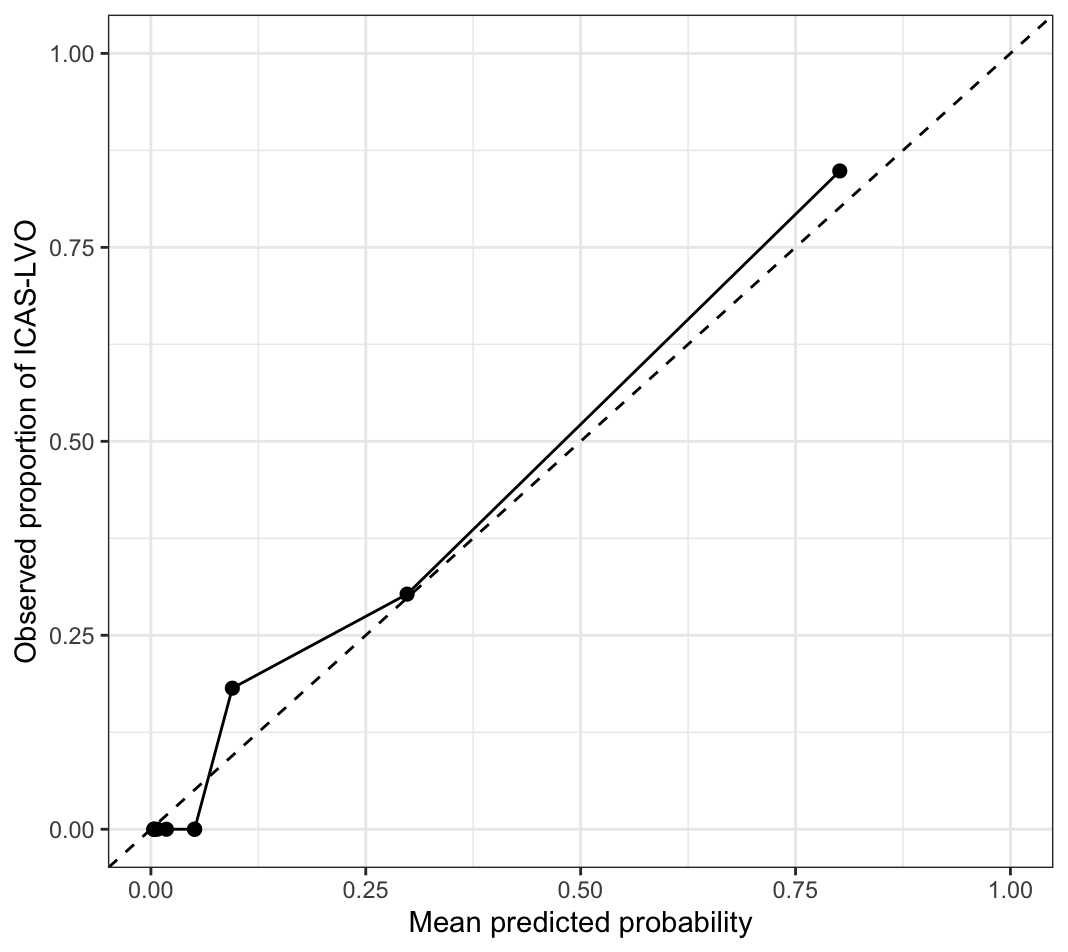


**Supplementary Figure 2.** Calibration of the ICAS‑M score for prediction of ICAS‑LVO.

The plot shows observed versus predicted probabilities of ICAS‑LVO across deciles of predicted risk according to the ICAS‑M score. The solid line represents the calibration of the fitted logistic regression model, and the dashed line represents the line of identity (perfect calibration). The calibration intercept and slope were approximately 0 and 1, respectively, indicating good agreement between predicted and observed risks.

ICAS-M, intracranial atherosclerotic stenosis model score; ICAS-LVO, intracranial atherosclerotic stenosis-related large vessel occlusion; ICAS, intracranial atherosclerotic stenosis; LVO, large vessel occlusion


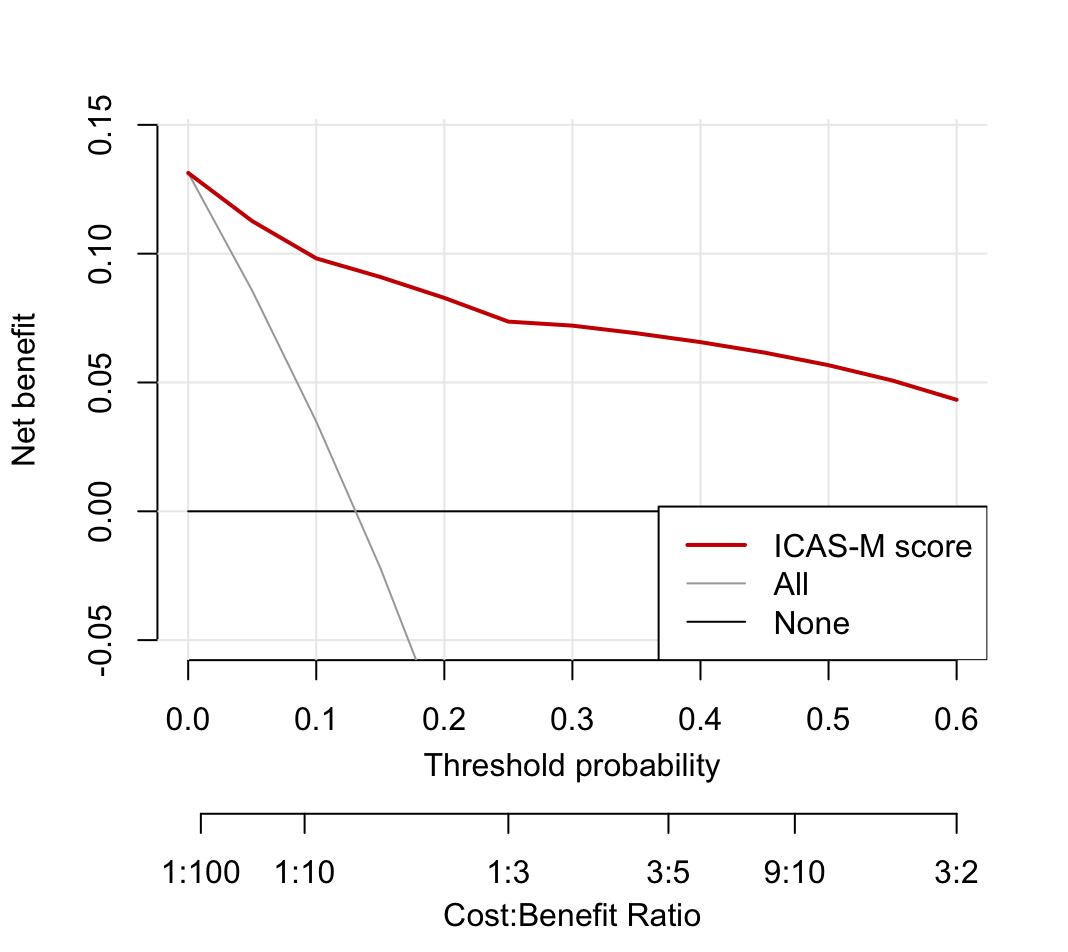


**Supplementary Figure 3.** Decision curve analysis for the ICAS‑M score.

The figure shows the net benefit of using the ICAS‑M score to predict ICAS‑LVO across a range of threshold probabilities, compared with default strategies of treating all patients or treating no patients. The ICAS‑M–based strategy yields higher net benefit than either “treat-all” or “treat-none” approaches over clinically relevant threshold ranges, thus supporting its potential clinical utility in MRI‑capable centers.

ICAS-M, intracranial atherosclerotic stenosis model score; ICAS-LVO, intracranial atherosclerotic stenosis-related large vessel occlusion; ICAS, intracranial atherosclerotic stenosis; LVO, large vessel occlusion; MRI, magnetic resonance imaging.
