# Supplementary material for: Magnetic resonance imaging and clinical prediction of intracranial atherosclerotic large vessel occlusion in acute ischemic stroke treated with endovascular thrombectomy
Source: Front Neurol. 2026 May 22;17:1803264. doi: 10.3389/fneur.2026.1803264 (PMC13236524; doi:10.3389/fneur.2026.1803264)
Supplement: Supplementary file 2 [file Data_Sheet_1.docx]

|  | All patients | | ICAS-LVO | | Non-ICAS-LVO | | P value |
| --- | --- | --- | --- | --- | --- | --- | --- |
|  | N=335 | | N=44 | | N=291 | |  |
| Stroke subtypes |  |  |  |  |  |  | <0.001 |
| Cardioembolism | 215 | (64) | 0 | (0) | 215 | (74) |  |
| Large artery atherosclerosis | 44 | (13) | 44 | (100) | 0 | (0) |  |
| Undetermined cause of stroke | 73 | (22) | 0 | (0) | 73 | (25) |  |
| Determined cause of stroke | 3 | (1) | 0 | (0) | 3 | (1) |  |
| Time course, min |  |  |  |  |  |  |  |
| Onset to Door | 150 | (69–365) | 265 | (93–711) | 144 | (65–345) | 0.001 |
| Onset to Puncture | 216 | (139–439) | 310 | (198–975) | 205 | (132–402) | <0.001 |
| Door to Puncture | 66 | (56–79) | 71 | (56–88) | 66 | (56–78) | 0.120 |
| Puncture to Recanalization | 61 | (40–90) | 79 | (56–114) | 56 | (39–88) | 0.001 |
| Endovascular thrombectomy |  |  |  |  |  |  |  |
| TICI ≥2b | 256 | (78) | 27 | (66) | 229 | (80) | 0.045 |
| TICI 3 | 147 | (45) | 17 | (41) | 130 | (45) | 0.738 |
| Endovascular passes | 2 | (1-2) | 2 | (1-2) | 2 | (1-3) | 0.956 |
| Complications of endovascular thrombectomy |  |  |  |  |  |  |  |
| Subarachnoid hemorrhage | 39 | (12) | 2 | (5) | 37 | (13) | 0.135 |
| Embolism in new territory | 5 | (1) | 0 | (0) | 5 | (2) | 1.000 |
| Extravasation | 5 | (1) | 1 | (2) | 4 | (1) | 0.508 |
| Intracranial hemorrhage |  |  |  |  |  |  |  |
| Parenchymal hemorrhage type I or II | 46 | (14) | 4 | (9) | 42 | (14) | 0.481 |
| Parenchymal hemorrhage type II | 12 | (4) | 2 | (5) | 10 | (3) | 0.662 |
| Outcomes |  |  |  |  |  |  |  |
| mRS score ≤ 2 at 3-month | 105 | (31) | 18 | (41) | 87 | (30) | 0.322 |
| Death at 3-month | 41 | (12) | 2 | (5) | 39 | (13) | 0.073 |

Supplementary table 1 Stroke subtypes, time courses, the outcomes of endovascular thrombectomy, and the clinical outcomes in patients with ICAS–LVO and Non–ICAS–LVO groups.

mRS indicates modified Rankin scale.

| **Predictors** | **β coefficient** | **OR (Firth)** | **95% CI** | **p value** |
| --- | --- | --- | --- | --- |
| Absence of AF | 2.80 | 16.51 | 5.25–67.76 | <0.001 |
| Multiple cortical/border-zone infarcts | 2.31 | 10.04 | 3.46–30.63 | <0.001 |
| Mixed acute–subacute infarcts | 1.56 | 4.75 | 1.47–15.82 | 0.010 |
| Absence of SVS | 1.47 | 4.33 | 1.75–11.28 | 0.002 |

Supplementary Table 2 Predictors of ICAS-LVO in Firth’s penalized logistic regression model.

AF, atrial fibrillation; OR, odds ratio; CI, confidence interval; SVS, susceptibility vessel sign.
